# More Than a Role Dispute: Physician Associates and the Changing Grounds of Medical Authority

**DOI:** 10.1111/1467-9566.70250

**Published:** 2026-08-13

**Authors:** Louise Ashley

**Affiliations:** ^1^ School of Business and Management Queen Mary University of London London UK

**Keywords:** interpretative repertoires, legitimacy, medical power, physician associates, workforce reform

## Abstract

This article examines how the legitimacy of physician associates (PAs) is constructed and contested in English medicine. Drawing on Critical Discursive Psychology, it analyses formal submissions to a government commissioned independent review of medical associate professions from the British Medical Association, General Medical Council and United Medical Associate Professionals to ask: What does conflict over PAs reveal about fractures in medical status and power? The findings show that these actors do not simply disagree about a new occupational role but mobilise shared concerns, especially around safety, standards and responsibility, through competing legitimacy logics. For the BMA, legitimacy is framed predominantly through boundary protection and the exclusivity of medical expertise; for the GMC, through regulatory governance and system assurance; and for UMAP, through workforce contribution and professional integration. Conceptually, this article extends jurisdictional accounts by showing that contemporary role disputes concern not only who controls clinical tasks but also which criteria and institutions are authorised to validate healthcare work. The PA controversy therefore reveals a pluralisation and institutional fragmentation of the grounds of medical authority, rather than its straightforward decline or reproduction.

## 1 Introduction

Physician associates (PAs) have become one of the most contested workforce roles in contemporary healthcare in England. PAs were introduced in the United Kingdom in the early 2000s to improve service capacity and continuity of care in high‐demand areas such as primary and emergency medicine. Trained to undertake diagnostic and treatment tasks under the supervision of doctors, PAs have been positioned as a workforce innovation intended to address persistent staffing shortages within the National Health Service (NHS) (Drennan et al. 2015, 2017, 2019a, 2019b; Halter et al. 2013). Early evaluations reported high levels of patient satisfaction and evidence of clinical safety, particularly where role boundaries and supervisory arrangements were clearly defined (Williams and Ritsema 2014). However, recent policy developments have intensified controversy surrounding the role. In particular, the introduction of formal statutory regulation of PAs by the General Medical Council (GMC) from December 2024 has generated polarised responses. Today, the legitimacy of PAs is contested across professional, institutional and public arenas, in debates sometimes described as ‘toxic’ (Abbasi 2023).

Controversies surrounding PAs can be approached as disputes over the legitimacy of a specific emergent occupational role. However, this article contends that it is equally productive to consider this as a site through which wider tensions over authority and professional boundaries within medicine are being articulated, hence the research question addressed here: *What do institutional contestations over the legitimacy of physician associates reveal about fractures in medical status and power in England?*

To address this question, this article analyses formal submissions made to the independent Leng Review of medical associate professions (MAPs), commissioned by the UK Department of Health and Social Care in 2024. The Leng Review provided a formal arena in which organisations were required to articulate and justify their positions on the legitimacy, regulation and future of these roles. The final report was published in July 2025, but the focus here is not on policy outcomes. Instead, it is on public submissions to the review made by three key actors, selected because they occupy important but also distinct positions within the field of medicine in England: the General Medical Council (GMC), as regulator; the British Medical Association (BMA), as representative body for doctors; and United Medical Associate Professionals (UMAP), as a representative organisation for PAs.

These submissions are read as institutional texts: public documents through which these organisations sought to frame the problem, attribute responsibility and advance particular understandings of professional authority, patient safety and workforce reform. Critical Discursive Psychology (CDP) (e.g., Potter and Wetherell 1987) is deployed as the key analytical tool, as this treats discourse not simply as descriptive but as a form of social action. The analysis pays particular attention to the interpretative repertoires (IRs) drawn upon by each organisation, understood as the recurring and culturally familiar ways of characterising PAs, through which different institutional accounts are made persuasive and authoritative. This approach is suited to this study because the central interest is not whether claims about PAs made by different institutions are ‘true’ or ‘false’ but how the legitimacy of this role is discursively constructed, defended and challenged in this contested policy arena.

The analysis shows that although the three institutional actors frequently invoke similar themes, such as patient safety, professional standards and workforce capacity, they mobilise these themes through strikingly different repertoires. For the BMA, PAs are framed primarily in relation to patient safety risk and threat to professional boundaries; for the GMC, as a role requiring regulatory oversight within a multiprofessional system; and for UMAP, as a workforce contribution requiring greater integration and recognition.

These differences go beyond expected disagreements between organisations with distinct interests or constituencies to suggest a form of discursive divergence in which shared concerns are mobilised through differently weighted legitimacy logics. Although not wholly incompatible, these logics reflect and arguably reproduce significant tensions over who is entitled to define safe, proper and legitimate healthcare work. The central argument advanced here is that the controversy concerns not only resistance to or acceptance of PAs but also the criteria and institutional sites through which clinical authority is validated. It therefore provides a novel and important window onto the fragmentation and reworking of medical authority across professional, regulatory, epistemic and symbolic dimensions (e.g., Ferguson et al. 2021). Before expanding on these findings and discussing implications for theory and practice, the following sections describe the theoretical framework in more detail and the research design.

## 2 Literature Review: Professional Legitimacy, Boundary Disputes and Transformations in Medical Authority

Long‐standing literature in the sociology of the professions offers several ways of understanding how professions secure status and power, though three traditions are especially relevant here. Freidson (1970) emphasised the structural foundations of medical dominance, arguing that medicine's authority rested on its alignment with state institutions, which historically granted the profession a high degree of autonomy, exclusive jurisdiction over core areas of healthcare work, and substantial control over credentialling and labour divisions. From this perspective, professional power is secured not simply through expertise but through institutional arrangements that protect medicine's privileged regulatory position.

Abbott (1988, 2005) foregrounded jurisdictional competition within a ‘system of professions’. In this framework, professions compete for control over tasks by asserting epistemic authority and demonstrating competence in particular domains of knowledge and practice. Jurisdictional disputes arise when emerging occupations seek to claim aspects of work previously controlled by an established profession or when established professions seek to defend or reclaim contested areas of expertise. In these struggles, knowledge claims and technical expertise become key resources through which professions seek to maintain or extend their boundaries.

Neo‐Weberian analyses emphasise the symbolic and status dimensions of professional ‘closure’. Larson (1977) is particularly influential here, having argued that professions function as status groups using structural and cultural strategies to exclude rivals and maintain distinction. Witz's (1992) concept of ‘demarcationary’ strategies is also useful, as it highlights how professions may assert distinction through discursive boundary work when traditional forms of exclusion, such as licensing or credentialling, are constrained or contested.

Together, these approaches suggest that professional power is secured not only through formal jurisdiction or technical knowledge but also through the capacity to define legitimate work, legitimate workers and legitimate relationships between occupations. Medicine has historically been one of the most successful examples of this capacity to define legitimate healthcare work and its boundaries. Parsons (1951), for example, portrayed the medical profession as occupying a privileged position grounded in norms of altruism and professional responsibility, with physicians expected to place patient welfare ahead of personal gain.

During the postwar period, this authority was said to have been reinforced by strong professional organisations and relatively stable institutional arrangements, producing what has sometimes been described as the ‘golden age of doctoring’ (McKinlay and Marceau 2002). Willis' (1989) account of medical dominance is useful here because it shows how medicine has historically organised the healthcare division of labour by controlling its own work, exercising authority over other occupations and defining the terms on which other groups may be recognised as legitimate. This includes attention to factors such as titles, training, knowledge base, supervision and the risk of misleading the public about occupational status. Saks' (2003) work on orthodox and alternative medicine similarly shows that boundary disputes in healthcare are fought not only over tasks but also over the authority to classify forms of practice as legitimate, scientific, safe and publicly trustworthy.

Together, this work directs attention beyond whether an emergent occupation is accepted or rejected, towards the conditions under which recognition is made possible. In this sense, disputes over PAs can be read as part of this longer history in which medicine has managed adjacent occupations through strategies of subordination, exclusion or incorporation, whereas newer groups seek recognition within or against these constraints. This is particularly salient because, from the 1970s onwards, scholars began to identify pressures that appeared to challenge this dominance. These included rising healthcare costs, greater government oversight, managerialism, patient consumerism, evidence‐based medicine and the expansion of pharmaceutical and biotechnology industries, all of which reshaped the conditions under which medical authority was exercised (Hafferty and Light 1995; Timmermans and Kolker 2004; Conrad 2005; Light 2010). Especially important for this study, however, is the professionalisation and expansion of other healthcare occupations, whose members increasingly sought recognition, autonomy, extended scopes of practice and jurisdictional legitimacy (Nancarrow and Borthwick 2005; Weiss and Sutton 2009; McMurray 2011).

Some scholars have argued that against this backdrop, medicine has experienced a gradual erosion or transformation of its traditional authority, though others cautioned against overstating this decline. Timmermans and Oh (2010, S94), for example, have argued that it may be ‘too early to count out the medical profession’, pointing to its historical resilience and capacity to adapt to institutional change. More recently, Epstein and Timmermans (2021) have reframed this debate by distinguishing between the social authority of physicians and the cultural authority of medicine, arguing that the latter has proliferated and diversified, shifting in part from medicine into broader terrains of health. This avoids a binary decline‐or‐continuity account of medical authority while raising questions around where authority is claimed to reside: in professional control over boundaries, regulatory systems of assurance or claims about workforce contribution and integrated care.

Although there is no consensus here, what is clear is that the landscape in which such contests take place has changed, with medical power and authority increasingly shaped by managerialism, external regulation, audit, accountability and pluralised claims to expertise (Evetts 2006, 2011; Noordegraaf 2011, 2020). Under such conditions, legitimacy, defined here as the symbolic and institutional recognition of a role as trustworthy, competent and appropriate within a professional domain (Suchman 1995), remains a crucial resource, but one which must be continually rearticulated in relation to multiple audiences and institutions.

It is within this changing context that emerging practitioner roles can become focal points for wider struggles over authority, jurisdiction and professional identity (McDermott et al. 2023). Scholarship has examined transformations within the medical profession through the lenses of workforce reform and the introduction of nonmedical practitioner roles (Currie et al. 2009; Thompson and McNamara 2022; Timmons et al. 2023). Workforce reform can introduce new forms of oversight, standardisation and organisational control, reducing the capacity of any single profession to unilaterally define and regulate its own work (Currie et al. 2009; Evetts 2006; Noordegraaf 2011). Such reforms do not simply redistribute tasks. They can also unsettle the symbolic and institutional arrangements through which professional authority is recognised and maintained.

PAs are a particularly useful case through which to examine these dynamics because they sit at the intersection of workforce reform, professional boundary work and institutional contestation. Research on PAs in England has already examined their deployment, acceptability, contribution to service delivery and effects on professional boundaries. Important work by Drennan et al. (2015, 2017, 2019a, 2019b) shows that PAs may support continuity of care, patient experience and team functioning, particularly where supervision and role clarity are in place, while also unsettling established professional relationships. However, less attention has been paid to how PA legitimacy is constructed in public institutional discourse, especially in the recent context of statutory regulation and intensified professional opposition.

This study addresses this gap by examining the discursive and institutional struggle over the terms on which the role is recognised as professionally acceptable. This distinction is conceptually important: Existing jurisdictional approaches illuminate competition over tasks and domains of work, whereas this study additionally asks how institutional actors define the criteria and institutional locations through which claims to clinical authority are validated.

International experience with similar roles illustrates that these developments do not unfold uniformly. In the United States, where the profession originated in the 1960s in response to physician shortages, the role has historically been known as ‘physician assistant’, though some professional bodies and jurisdictions have moved towards ‘physician associate’ terminology. In Canada, ‘physician assistant’ remains in use. Such variations matter because titles help negotiate role status and public intelligibility. In both countries, early concerns around supervision, accountability and scope of practice appear to have softened as regulatory frameworks clarified responsibilities and PAs demonstrated value within supervised models of care (Mittman et al. 2002; Hooker and Everett 2012; Hooker and Muchow 2015; Contandriopoulos et al. 2016).

England, and the United Kingdom more widely, presents a different institutional setting, where workforce shortages, waiting lists, pay erosion and dissatisfaction among junior, or ‘resident’, doctors have contributed to a highly visible debate about professional status and workforce reform (NHS England 2023; House of Commons Library 2024; General Medical Council 2024a). PAs have been promoted as one mechanism for redistributing clinical tasks within medical teams, as generalist healthcare practitioners who usually complete a 2‐year postgraduate PA programme combining theoretical and clinical practice components. PAs work under the supervision of a named senior doctor, with scope of practice determined locally (NHS Employers 2025). By late 2024, there were over 3500 PAs working in NHS England, whereas the GMC estimated around 5000 across the United Kingdom (NHS England 2024; General Medical Council 2024b). PAs have been regulated by the GMC since 2024, prior to which the faculty of physician associates held a managed voluntary register, which closed to new entrants once the GMC became the regulator (Royal College of Physicians 2024). This reform was therefore institutionally significant, as a regulator historically associated with doctors extended its remit to MAPs, introducing registration requirements, standards and oversight arrangements.

Related controversies informed the subsequent ‘Leng Review’ (Department of Health and Social Care 2025), submissions to which comprise the dataset here. These submissions were formal contributions to an official review process, requiring actors to articulate and justify their positions within a shared policy and regulatory arena. They therefore represent a distinctive genre of institutional communication: public‐facing, formal documents designed explicitly to influence regulatory and policy deliberations. This offers a particularly valuable window into how organisations articulate legitimacy claims in a setting where professional authority, expertise and institutional credibility must be carefully performed, with data collection and analysis explained in further detail next.

## 3 Methods: Data Collection—Public Submissions to the Leng Review

Although submissions to the Leng Review were made by a variety of institutions, including Royal Colleges and bodies such as the United Kingdom's Care Quality Commission, the analysis here focuses on three actors: the British Medical Association (BMA), representing doctors; United Medical Associate Professionals (UMAP), representing physician associates; and the General Medical Council (GMC), the profession's statutory regulator, responsible for setting professional standards and overseeing registration and professional conduct.

These organisations occupy central but distinct positions within the institutional field, making them the most relevant actors through which to examine how legitimacy is discursively constructed and contested. This approach also allows a more detailed examination and presentation of discursive strategies and comparative analysis without compromising analytical brevity. The BMA's submission (British Medical Association 2025) is 17 pages in total, whereas the General Medical Council’s (2025) submission is 11 pages. UMAP submitted a cluster of materials covering both anaesthesia associates (AAs) and PAs (UMAP 2025a, 2025b, 2025c, 2025d, 2025e, 2025f, 2025g, 2025h, 2025i). Although many of the issues discussed in the Leng Review also relate to anaesthesia associates (AAs), the analysis here focuses on PAs, who have attracted substantially greater public, professional and media attention and therefore offer a particularly visible site of legitimacy contestation.

All documents analysed were publicly available, but ethical considerations were carefully observed through a transparent analytic approach and careful interpretation of institutional positions. Importantly, the focus on discourse does not imply that concerns raised by organisations about the PA role are merely rhetorical or lacking substantive grounding. Rather, the analysis proceeds from the premise that actors draw upon particular linguistic resources because they are strategically useful within specific institutional contexts (Swidler 1986). The aim is therefore not to adjudicate the ‘truth’ or validity of competing claims but to examine how legitimacy is constructed and made persuasive through discourse.

### 3.1 Analytical Approach—Using the Critical Discursive Psychology Approach

Although several approaches to discourse analysis are available, this study uses Critical Discursive Psychology (CDP) because it treats discourse as action‐oriented and rhetorically organised, attending to how descriptions are assembled to appear factual, reasonable and authoritative rather than as neutral reflections of an underlying reality (Billig 1987; Potter and Wetherell 1987). This makes CDP especially suitable here because the analytic interest lies not simply in the substantive positions taken in the submissions, but in how those positions are constructed, justified and made persuasive, and in what those constructions accomplish. CDP is also particularly useful for examining public policy submissions because such texts are explicitly argumentative, audience‐aware and oriented to questions of accountability, credibility and stake.

A central analytic concept within this approach is tha't of interpretative repertoires: relatively familiar and recurrent ways of describing actors, problems and solutions that can be traced across texts and used to identify broader, more stable argumentative logics (Wetherell and Potter 1988). Their value here is that they make it possible to move beyond isolated claims to identify the wider logics through which institutions rendered the PA role intelligible and more (or less) legitimate.

One way to identify such repertoires is to identify the local discursive features of texts. Drawing from related literature (e.g., Potter and Wetherell 1987), this study used four key headings: claim function, to identify what a passage was doing argumentatively; rhetorical strategy, to capture the multiple devices through which claims were made credible or less contestable; tone, to register differences in style and institutional register; and evaluative stance, to identify how actors such as PAs, doctors and regulators were positioned normatively, for example as risky, defensible or problematic.

The analysis took place in three main stages. First, initial repeated close readings of submissions led to the production of multiple codes under each of the four headings introduced above. Working iteratively between theory and data, these codes were gradually refined via repeated reading of the submissions, resulting in the framework depicted in Table 1.

**TABLE 1:** Final coding framework.

Analytical dimension	Final codes	Purpose
Claim function	Criticising, defending, warning, clarifying, evidencing, explaining, recommending	What the segment is doing argumentatively
Rhetorical strategy	Anecdotal evidence, authority appeal, categorical distinction, contribution, evidence claim, institutional blame, jurisdiction claim, risk amplification, risk management	How claims were made persuasive, credible or difficult to contest
Tone	Technical, corrective, urgent, neutral	Differences in register and style
Evaluative stance	Critical, supportive, neutral, ambivalent	How actors, roles or arrangements were positioned normatively

Second, each submission was coded in full, using this framework, and working section by section, at the level of sentences or short passages that articulated identifiable claims about PAs and related issues. Claim function, tone and evaluative stance were each coded once per segment, though more than one rhetorical strategy could be assigned to the same segment, recognising that institutional arguments are often layered, with the same passage performing several persuasive moves at once. The rhetorical strategy codes were especially informed by the CDP concern with how accounts are made persuasive and difficult to contest and developed inductively through repeated reading of the submissions. For example, passages invoking survey data, formal reports or numerical findings were coded as evidence claim; passages grounding legitimacy in institutional remit, legal authority, expertise or standards as authority appeal; passages emphasising harm, public confusion, unsafe substitution or preventable danger as risk amplification; and passages presenting risk as manageable through supervision, standards, assessment or oversight as risk management.

This process resulted in a single spreadsheet in which coded segments from all three submissions were organised row by row. During the third phase of analysis, the author engaged in comparison within and across submissions, using this spreadsheet to identify recurring combinations of themes, strategies, tones and evaluative positions which clustered together into broader argumentative patterns for each institution to form specific interpretative repertoires (IRs).

Because institutional texts rarely proceed through an entirely uniform logic, more than one repertoire was identified in each submission. However, the analysis sought to identify the dominant repertoire in each, judged partly by recurrence, though more heavily by the extent to which a repertoire organised the central argumentative thrust of the submission and incorporated the widest and most stable cluster of associated claims, strategies, tones and evaluative positions. As with qualitative discourse analysis generally, the aim was not statistical generalisability or replicability, but analytic defensibility. Validity rested on repeated checking of coding decisions against the full texts, iterative comparison within and across submissions and a transparent audit trail linking first‐order coding to higher‐order interpretative claims.

## 4 Findings

This section identifies and explains the dominant interpretative repertoire through which each actor constructs the legitimacy of PAs.

### 4.1 BMA: Patient Safety Through Boundary Protection

The dominant interpretative repertoire in the BMA submission can be characterised as *patient safety through boundary protection*. This repertoire was identified through the repeated clustering of evidencing, warning and criticising claim functions around questions of patient safety, insufficient training, misleading titles, public confusion, unsafe substitution and weaknesses in existing arrangements. These issues constantly recurred across the submission, contributing to a broader argumentative pattern in which PAs are made intelligible as a source of risk because they blur the distinction between medically qualified doctors and non‐doctor practitioners. As such, the submission not only raises opposition to PAs at the level of policy preference but also makes uncertainty about status, competence and scope inseparable from questions of public protection.

With respect to rhetorical strategies, this repertoire is sustained through recurring use of evidence claim, authority appeal, risk amplification, institutional blame, categorical distinction and jurisdiction claim. Using these strategies, this BMA is able to present its position as a necessary response to institutional arrangements, which are represented as unsafe, rather than an explicit defence of professional interest, which might be read by external audiences as illegitimate. The dominant tone across the submission is corrective, shifting at key moments into urgent, whereas the evaluative stance is consistently critical. The cumulative effect is to move the debate away from whether a new occupational role might contribute to service delivery and towards whether existing distinctions between levels of clinical authority have been eroded in ways that expose patients to harm.

A central theme within this repertoire is the repeated insistence that PAs are not doctors and should not be mistaken for them, especially clear in the BMA's opening discussion of terminology, where it states that PAs ‘are not medical practitioners’, ‘are not medically trained’ and ‘cannot be described as medical professionals’ (p. 2). On one level, these formulations are merely descriptive. Arguably, however, using strategies including categorical distinction and jurisdiction claim, they function to stabilise a hard boundary around legitimate medical authority by tying it to qualification and, by implication, the exclusivity of medical expertise. The issue moves, then, beyond role differentiation in an administrative sense to also highlight the preservation of a symbolic and practical distinction on which professional legitimacy depends.

This boundary work is enforced through repeated evidence claim and authority appeal, used to suggest that the distinction between doctors and PAs is not being maintained in practice. One example is the BMA's reference to its omnibus survey of over 2000 members of the public conducted in November 2023, which found that ‘one in four’ respondents believed PAs were doctors, whereas only 46% said it was always clear when they were being treated by a doctor (p. 2). Although survey findings are foregrounded here, the significance of this material lies particularly in what these findings are made to do: In short, public misunderstanding is presented as *evidence* that existing institutional arrangements have failed to preserve the visibility and recognisability of medical *authority*. Institutional blame is also important here because confusion is not framed as accidental or isolated but as the consequence of wider failures in naming, regulation and role design. Boundary defence is therefore recast as a matter of patient safety rather than one of occupational closure.

In places, the submission intensifies these claims through risk amplification. This is especially evident in its discussion of the tragic death of Emily Chesterton in 2022, attributed to an alleged misdiagnosis by an a physician associate. This case is presented in the BMA's submission alongside findings from two coroners: that the term ‘Physician Associate’ is misleading to the public and that the combination of an indistinct uniform and the title ‘Physician’ gives rise to confusion as to whether the practitioner is a doctor (p. 2). The case functions rhetorically by condensing several strands of the argument into a single urgent example: ambiguous title, public misunderstanding, insufficient distinction and serious harm. The dominant tone shifts in this context from corrective to urgent, whereas the evaluative stance remains strongly critical. One effect is that the issue is moved beyond one of implementation and is framed instead as both morally urgent and preventable.

Overall, these recurring features point to a coherent argumentative logic, where PAs are differentiated from doctors in categorical terms; institutions are criticised for failing to preserve that distinction; and this failure is represented as generating confusion, unsafe substitution and risk to patients. The BMA's submission goes well beyond criticism of a workforce role to provide a broader account in which safe care is made dependent on maintaining firm boundaries around medical expertise. In this respect, the BMA submission simultaneously challenges the legitimacy of PAs as an emergent occupational group while also defending a particular basis of legitimacy for medicine itself. Legitimate clinical authority is located in the *exclusivity* of medically qualified expertise, whereas role expansion is rendered problematic insofar as it appears to dilute, obscure or displace that authority. In this way, the controversy over PAs becomes a site through which wider anxieties about the erosion of medical distinctiveness, professional control and status are articulated through the language of public protection.

### 4.2 GMC: Regulatory Assurance and System Governance

The dominant interpretative repertoire in the GMC submission is *regulatory assurance and system governance*. This repertoire was identified through the repeated clustering of explaining, clarifying, evidencing and recommending claim functions around issues including statutory regulation, standards, registration, oversight, revalidation and public protection. Unlike the BMA, which constructs the PA role primarily through the problem of boundary erosion, the GMC treats PAs as workforce roles whose legitimacy depends on being governed within a robust regulatory framework. Legitimacy is therefore located in systems capable of defining standards, verifying competence and maintaining oversight, rather than professional exclusivity, and regulation is presented as the primary mechanism through which safe and legitimate practice is made possible.

This repertoire is sustained primarily through recurring use of authority appeal and risk management rhetorical strategies, in relation to which the GMC repeatedly invokes its statutory remit, legal authority, formal standards and regulatory procedures to present the PA role as governable. Across the submission, the dominant tone is consistently neutral, as is the evaluative stance. With respect to the latter, the submission generally avoids overtly positive or negative normative positioning of PAs, tending instead to frame the issue in procedural and administrative terms. One implication is that controversy is translated into a less emotive question about whether appropriate systems of assessment, monitoring and accountability are in place, in the process shifting the basis of legitimacy away from who PAs are, in relation to doctors, and towards whether the regulatory architecture is able to assure safe practice.

These themes are especially clear in the GMC's account of its own authority. For example, the submission states that ‘We decide, in line with the legal requirements in the AA PA Order, who can work as a PA or AA by setting the standards and requirements that individuals must meet to enter our register’ (p. 4). Similar formulations recur in the discussion of course approval, where education providers ‘must apply for approval of their course’ and programmes must ‘meet our standards’ to secure or maintain approval (p. 4). Authority appeal is central here, as the GMC grounds legitimacy in its legal remit and standard‐setting role. Superficially, these statements describe an administrative process, but they do so in terms which position the GMC as the institution specifically entitled to define admissibility, competence and the conditions of safe entry into practice. Authority, therefore, is positioned as resting in regulatory jurisdiction, rather than occupational status.

A second important rhetorical feature of the submission is its recurring use of the risk management rhetorical strategy, in relation to which the GMC does not seek to deny risk but to make it procedurally manageable. For example, the GMC explains that once courses are approved, they ‘enter our rolling cycle of quality assurance to ensure that they maintain our standards’ and that it undertakes ‘both proactive and reactive quality assurance action’ to monitor compliance (p. 4). Similarly, the submission notes that the Order requires practitioners to be assessed periodically ‘through the process of revalidation’ (p. 8), with PAs and AAs expected to provide evidence at appraisal that they ‘remain competent to undertake their role’ and are ‘up to date and fit to practise’ (p. 9).

This same logic is apparent in the GMC's discussion of registration assessments and professional standards. Registration assessments, for example, are said to ‘assure us that eligible PAs and AAs have the right clinical knowledge and skills’ and to ‘set a common threshold for safe practice’ (p. 5). Elsewhere, the GMC argues that ‘regulation is already beginning to raise standards of practice’ (p. 7), noting that some applicants were not granted registration because they had not passed the relevant examination. The submission also underlines that doctors, PAs and AAs ‘must follow Good medical practice’ (p. 5), whereas concerns are assessed against the GMC's ‘overarching objective to protect the public’, including ‘promoting and maintaining public confidence in the medical professions’ (p. 6). Across these examples, patient safety is framed as the outcome of standards, oversight and ongoing review.

Overall, the argumentative logic generated by these features follows a pattern in which workforce roles are said to exist; regulation provides oversight; standards and assessments establish competence; and ongoing monitoring helps maintain safe practice. The GMC therefore articulates a different basis of legitimacy from that of the BMA, with authority resting less on professional boundary maintenance and more on standards, oversight and the capacity of regulatory systems to make practice visible, assessable and correctable. In this sense, the submission points to a wider tension within contemporary medicine, between professional and regulatory authority, and to the possibility that legitimacy may be increasingly located in governance rather than professional exclusivity.

### 4.3 UMAP: Workforce Contribution and Professional Integration

The dominant interpretative repertoire in the UMAP submission is characterised as *workforce contribution and professional integration*. This repertoire was identified through the repeated clustering of evidencing, clarifying, defending and recommending claim functions around service delivery, multidisciplinary teamwork, supervision, role clarity and workforce need. Across the multiple texts which comprise UMAP's submission, PAs are represented as contributors to service delivery, as embedded members of multidisciplinary teams, as safely supervised practitioners and as professionals whose effectiveness depends on clearer pathways, stability and recognition. UMAP does not construct PAs primarily as a problem to be contained or as a category requiring strict limitation. Rather, it presents them as a legitimate occupational group whose value lies in what they contribute to patient care and how they can be incorporated safely and coherently into existing systems of practice. Legitimacy is therefore located in demonstrated usefulness, supervised competence and institutional integration.

In terms of associated rhetorical strategies, this repertoire is sustained through recurring use of contribution, evidence claim, risk management, authority appeal and anecdotal evidence. The dominant tone is technical, whereas the evaluative stance is predominantly supportive, though with some ambivalent passages where criticism or controversy is acknowledged. Unlike the BMA, UMAP does not make boundary maintenance the condition of safe care, and, unlike the GMC, it does not rest its case primarily on the authority of regulation. Instead, it seeks to ensure the PA role is persuasive by showing it *already* delivers value in practice and that it can do so safely, assuming it is properly integrated. This approach shifts the grounds of legitimacy towards contribution, so that the role is justified not because it resembles medicine, nor because it is governable, but because it is *already* useful within team‐based care.

This is especially clear in UMAP's repeated use of the contribution strategy. In its public summary of evidence submitted to the review, UMAP states that MAPs ‘provide safe, effective, and high‐quality care, while supporting NHS stability amid ongoing workforce shortages,’ and that PAs are ‘critical in ensuring continuity of care’ (UMAP 2025h). Benefit and service contribution are underlined here, and the role is additionally justified through what it enables: continuity, capacity and support for overstretched services. UMAP's discourse might then be read as an attempt to convert functional usefulness into institutional and professional recognition, to support an underlying argument where contribution should count as a legitimate basis of authority within contemporary healthcare.

UMAP makes the role additionally credible by drawing on claims about everyday practice, supervisor experience, patient evaluation and measurable organisational impact, using strategies including evidence claim, authority appeal and anecdotal evidence. Again, drawing on UMAP's (2025h) summary as illustration, it argues here for example, that ‘supervisors, patients, and workforce planners have long understood’ that MAPs are safe and effective and that ‘Supervisor and patient feedback consistently rates MAPs as ‘Very Good’ across all clinical domains.’ It notes that in one NHS trust, PA contributions saved ‘over 300 hours of junior doctor time each week through discharge support alone.’ The PA role is presented as an *already* functioning part of service delivery whose value is visible in practice, dismissing alternative arguments that these reforms are speculative and contingent.

A further important feature of the submission is its emphasis on integration, supervision and role clarity. UMAP does not deny the relevance of patient safety. Critically, however, safety is not framed as incompatible with the PA role, as with the BMA, but is positioned as achievable when properly embedded within multidisciplinary teams. The necessity for close supervision is therefore situated as part of the normal conditions through which safe contribution is secured, rather than evidence of deficiency. This logic is visible as UMAP (2025a) highlights the ‘critical role of supervision in ensuring safe and effective PA practice’ (p. 9) and underlines guidance that helps ensure ‘role clarity, appropriate supervision, and professional development opportunities’ (p. 10). Here, risk management is important because it allows safety to be presented as compatible with the role under appropriate conditions. As such, PAs are not presented as displacing doctors but as operating within a collaborative and supervised model of care in which clarity and safety are mutually reinforcing.

It is important to acknowledge a more defensive strand in UMAP submissions. For example, UMAP (2025h) argues that this is ‘not a debate grounded in evidence’ but one ‘driven by protectionism and power consolidation’ and that the campaign to misrepresent the role is ‘not only baseless but harmful to workforce morale, integration, and ultimately, to patient care’. However, this more adversarial element is secondary to the broader logic of contribution and integration and functions largely to protect the positive account already being advanced. Overall, contribution, supervision, team integration, role clarity and recognition are organised into a coherent repertoire in which legitimacy is constructed as available under conditions of safe integration, to suggest, in turn, that PAs already add value; that value can be evidenced in practice; safety depends on appropriate supervision and clearer role design; and stronger integration will allow the role to function more effectively and more visibly within the healthcare system.

Overall, the submission advances a model of legitimacy in which safe care does not have to depend on preserving professional exclusivity for doctors, to suggest that this new occupational group can be recognised and embedded within multidisciplinary practice. As an attempt to translate practical usefulness into institutional recognition and, ultimately, a more secure claim to authority, UMAP's argument might then be read as part of a broader professionalisation project. The key point here is that the controversy over PAs becomes a site through which an emergent occupation seeks to establish that contribution, not exclusivity, should count as a basis of legitimacy in contemporary medicine.

## 5 Discussion and Conclusion

This study has examined how key institutional actors in medicine in England discursively construct, defend and contest the legitimacy of physician associates and what this reveals about changing medical status and power. Its central finding is not simply that the BMA, GMC and UMAP disagree about the PA role, but that they organise similar concerns, especially safety, standards, responsibility, supervision and public protection, through only partly compatible legitimacy logics.

Critical here is that patient safety functions across all three submissions as a central (de)legitimating term yet does different discursive work in each. For the BMA, safety is tied to blurred boundaries, unsafe substitution and the erosion of distinctions between doctors and non‐doctors and is articulated through symbolic legitimacy, invoking public trust, role confusion and the moral distinctiveness of the doctor in a corrective and, at times, urgent register. Read through the framework provided by Willis (1989), the BMA submission can be understood not simply as opposition to PAs, but as an attempt to reassert medicine's authority to define the conditions under which an adjacent occupation may be recognised as legitimate. Its emphasis on misleading titles, public confusion, insufficient training and supervision, and unsafe substitution works to recast jurisdictional limitation as patient protection. For the GMC, safety is framed as something secured through standards, registration, oversight and ongoing review, with a particular emphasis on regulatory legitimacy and procedural language. For UMAP, safety is also represented as compatible with the PA role, provided supervision, integration and role clarity are in place, emphasising a form of pragmatic legitimacy, articulated through a supportive language of recognition and inclusion.

It is worth noting here that the distinction between the GMC and UMAP should not be overstated. In contrast to the BMA, both organisations treat the PA role as potentially legitimate under appropriate conditions of supervision, role clarity and governance. In this sense, their submissions are aligned around a broadly integrative model of workforce reform. Where they more obviously differ, on the other hand, is in the institutional position from which this integration is made persuasive. The GMC's account is primarily top‐down and regulatory, so that PAs are rendered legitimate insofar as they can be incorporated into formal systems of standards, registration, assessment and oversight. UMAP's account, by contrast, is bottom‐up and occupational, so that PAs are rendered legitimate through evidence of contribution, team embeddedness, continuity of care and the practical value they already bring to service delivery. The difference, then, is not that one organisation accepts PAs while the other rejects them but that each locates the grounds of legitimacy differently. For the GMC, legitimacy is conferred through a type of regulatory governability, whereas, for UMAP, it is claimed through demonstrated contribution and recognition within multidisciplinary practice.

That organisations with different interests and constituencies disagree is not, in itself, surprising. However, the specific contribution of this article is to show that competing institutional actors contest not only the allocation of healthcare work but also the criteria and institutional authorities through which claims to that work are validated. The divergence identified here points to a pluralisation and fragmentation in the basis of authority within contemporary medicine, shaped by the different institutional positions, audiences and pressures through which actors must perform legitimacy. Two fractures are especially visible: one between professional and regulatory authority and another between status protection and workforce pragmatism.

In one sense, these findings are consistent with classic accounts of jurisdictional conflict in the sociology of the professions (Abbott 1988, 2005). The BMA's defence of professional boundaries, UMAP's attempts to secure recognition for a newer occupational group and the GMC's position as regulatory arbiter can all be read in those terms and align with earlier research on PAs in England which suggests that the role challenges established professional boundaries (e.g., Drennan et al. 2017). Research on workforce reform has shown that such developments often emerge where existing professional settlements are already under strain (Currie et al. 2009; Drennan et al. 2015, 2019a, 2019b). However, jurisdictional analysis alone does not fully capture disagreement over the evaluative criteria and institutional authorities through which such claims are judged.

A more modest implication concerns the rhetorical registers through which institutional actors make these claims persuasive. The BMA occasionally intensifies and moralises its case, reporting that doctor substitution had been described to it as a ‘scandal’, characterising the GMC's approach as ‘highly irresponsible’ and invoking the ‘tragic case’ of Emily Chesterton. UMAP's more defensive language refers to ‘protectionism and power consolidation’ and to harm to workforce morale (UMAP 2025h), whereas the GMC relies predominantly on procedural language. These examples suggest that institutional position shapes the rhetorical register through which legitimacy claims are articulated, with representative and regulatory actors drawing on differently weighted discursive resources.

The PA case in England also goes beyond a straightforward jurisdictional dispute because what is at stake is not only competition over task or territory but also conflict over the grounds on which legitimacy itself is conferred. Here, the BMA locates legitimacy in the symbolic distinctiveness and exclusivity of the doctor; the GMC in the procedural authority of regulation, standard‐setting and oversight; and UMAP in demonstrated contribution to service delivery and safe supervised practice. The analysis therefore extends jurisdictional accounts by identifying a second‐order contest over the standards and institutional sites through which jurisdictional claims are validated. This is one sense in which the PA controversy illustrates what Epstein and Timmermans (2021) describe as the proliferation and diversification of cultural authority in health: Authority is not simply transferred away from medicine but reconstructed across professional, regulatory, organisational and occupational sites.

Overall, these differences can be understood as competing legitimacy logics through which medical work is ordered and justified. They may also explain why the controversy has become so intense because it concerns not simply the place of PAs, but the wider question of who has authority to define what counts as safe, proper and legitimate care, closely related to which are three competing models of healthcare authority. For the BMA, this is a professional monopoly model, in which doctors retain primary control over diagnosis, treatment and the boundaries of legitimate clinical work. For the GMC, it is a regulated multiprofessional model in which multiple occupations are authorised through common systems of governance. For UMAP, it is a workforce hybridisation model, in which flexible roles are integrated into multidisciplinary care and justified through contribution and supervised competence. Critically, these models are difficult to reconcile because they rest on different assumptions about who should control expertise, define safe care and organise clinical labour.

In making these points, several limitations of this study should be acknowledged. First, this study analyses formal institutional submissions to the Leng Review, which capture public, strategic and audience‐aware accounts rather than backstage deliberations or everyday clinical practice. Second, the focus on a limited number of prominent institutional actors was appropriate for analysing legitimacy claims in a bounded policy arena but necessarily excludes other important voices, including patients, employers, frontline clinicians and policymakers, which are only indirectly present here. This matters because legitimacy in healthcare is never conferred by professional actors alone. Third, the findings are shaped by the specific institutional and historical conditions of the United Kingdom, and the fractures identified here may not transfer straightforwardly to settings where PA‐like roles have different histories, scopes of practice or regulatory settlements. Future research could therefore examine how PA legitimacy is constructed and contested in clinical settings and by publics beyond institutional elites and whether comparable disputes in other healthcare systems reveal similar transformations in medical status and power.

Despite these caveats, the article's core contribution is to show that disputes over PAs concern not only the distribution of clinical work but also the grounds on which clinical authority is recognised. Medical authority emerges here not as a unitary possession that the profession either retains or loses, but as something constructed across professional, regulatory and occupational sites whose legitimacy claims only partly align. A key point is that such conflicts should not be understood only as zero‐sum struggles. Although the BMA, GMC and UMAP often appear to speak at cross‐purposes, common ground is not absent from the debate, albeit often obscured by institutional antagonism and the difficulty of co‐producing legitimacy across competing institutional logics. This opens an important sociological and political possibility, namely, that a more negotiated settlement over factors such as role clarity, accountability, supervision and safe practice need not further diminish medical authority. On the contrary, where professional authority is increasingly exercised under organisational and interdependent conditions, doctors may retain influence not only by *defending* boundaries but also by shaping the standards and coordinating arrangements through which new roles are governed (Evetts 2011; Noordegraaf 2020; Schot et al. 2020).

A final contribution for the sociology of health and illness is therefore to identify a pluralised legitimacy order in which professional qualification, regulatory assurance and demonstrated workforce contribution operate as overlapping but competing bases of clinical authority. Under contemporary conditions, doctors' status and power may depend less on preserving an uncontested monopoly and more on retaining the capacity to shape the standards, boundaries and coordinating arrangements through which legitimate clinical work is organised. In sum, the PA controversy therefore illustrates not the disappearance of medical authority, but its reconstitution through ongoing negotiation across professional, regulatory and organisational sites.

## Author Contributions

**Louise Ashley:** conceptualization, investigation, writing – original draft, methodology, validation, writing – review and editing, formal analysis, project administration, data curation.

## Funding

The author has nothing to report.

## Ethics Statement

All documents analysed were publicly available and did not involve human participants. As such, formal ethical approval was not required. However, ethical considerations were observed through a transparent analytic approach and careful interpretation of institutional positions, as explained in the submission.

## Consent

The author has nothing to report.

## Conflicts of Interest

The author declares no conflicts of interest.

## Data Availability

This paper is based on submissions to the Leng Review, which are publicly available, online, and are fully referenced.

## References

[shil70250-bib-0001] Abbasi, K. 2023. “The Debate Over Physician Associates Is Necessary—But Needlessly Toxic.” BMJ 383: p2821. 10.1136/bmj.p2821.

[shil70250-bib-0002] Abbott, A. 1988. The System of Professions: An Essay on the Division of Expert Labor. University of Chicago Press.

[shil70250-bib-0003] Abbott, A. 2005. “Linked Ecologies: States and Universities as Environments for Professions.” Sociological Theory 23, no. 3: 245–274. 10.1111/j.0735-2751.2005.00253.x.

[shil70250-bib-0004] Billig, M. 1987. Arguing and Thinking: A Rhetorical Approach to Social Psychology. Cambridge University Press.

[shil70250-bib-0005] British Medical Association . 2025. BMA Submission to the Independent Review of the Physician Associate and Anaesthesia Associate Professions. www.bma.org.uk/media/lgabqhbk/bma‐submission‐to‐the‐leng‐review‐april‐2025.pdf.

[shil70250-bib-0006] Conrad, P. 2005. “The Shifting Engines of Medicalization.” Journal of Health and Social Behavior 46, no. 1: 3–14. 10.1177/002214650504600102. 15869117

[shil70250-bib-0007] Contandriopoulos, D. , A. Brousselle , M. Breton , et al. 2016. “Nurse Practitioners, Canaries in the Mine of Primary Care Reform.” Health Policy 120, no. 6: 682–689. 10.1016/j.healthpol.2016.03.015. 27085958

[shil70250-bib-0008] Currie, G. , R. Finn , and G. Martin . 2009. “Professional Competition and Modernizing the Clinical Workforce in the NHS.” Work, Employment & Society 23, no. 2: 267–284. 10.1177/0950017009102858.

[shil70250-bib-0009] Department of Health and Social Care . 2025. Independent Review of the Physician Associate and Anaesthesia Associate Roles: Final Report. Department of Health and Social Care.

[shil70250-bib-0010] Drennan, V. M. , J. Gabe , M. Halter , S. de Lusignan , and R. Levenson . 2017. “Physician Associates in Primary Health Care in England: A Challenge to Professional Boundaries?” Social Science & Medicine 181: 9–16. 10.1016/j.socscimed.2017.03.045. 28364578

[shil70250-bib-0011] Drennan, V. M. , M. Halter , L. Joly , et al. 2015. “Physician Associates and GPs in Primary Care: A Comparison.” British Journal of General Practice 65, no. 634: e344–e350. 10.3399/bjgp15x684877. PMC4408498 25918339

[shil70250-bib-0012] Drennan, V. M. , M. Halter , C. Wheeler , et al. 2019a. “The Role of Physician Associates in Secondary Care: The PA‐SCER Mixed‐Methods Study.” Health Services and Delivery Research 7, no. 19: 1–158. 10.3310/hsdr07190. 31162917

[shil70250-bib-0013] Drennan, V. M. , M. Halter , C. Wheeler , et al. 2019b. “What Is the Contribution of Physician Associates in Hospital Care in England? A Mixed Methods, Multiple Case Study.” BMJ Open 9, no. 1: e027012. 10.1136/bmjopen-2018-027012. PMC6359738 30700491

[shil70250-bib-0014] Epstein, S. , and S. Timmermans . 2021. “From Medicine to Health: The Proliferation and Diversification of Cultural Authority.” Journal of Health and Social Behavior 62, no. 3: 240–254. 10.1177/00221465211010468. 34528483

[shil70250-bib-0015] Evetts, J. 2006. “Short Note: The Sociology of Professional Groups: New Directions.” Current Sociology 54, no. 1: 133–143. 10.1177/0011392106057161.

[shil70250-bib-0016] Evetts, J. 2011. “A New Professionalism? Challenges and Opportunities.” Current Sociology 59, no. 4: 406–422. 10.1177/0011392111402585.

[shil70250-bib-0017] Ferguson, J. , A. Tazzyman , K. Walshe , et al. 2021. “‘You're Just a Locum’: Professional Identity and Temporary Workers in the Medical Profession.” Sociology of Health & Illness 43, no. 1: 149–166. 10.1111/1467-9566.13210. 33112436

[shil70250-bib-0018] Freidson, E. 1970. Professional Dominance: The Social Structure of Medical Care. Atherton Press.

[shil70250-bib-0019] General Medical Council . 2024a. GMC Regulation of Physician Associates and Anaesthesia Associates to Begin. https://www.gmc‐uk.org/news/news‐archive/gmc‐regulation‐of‐physician‐associates‐and‐anaesthesia‐associates‐to‐begin.

[shil70250-bib-0020] General Medical Council . 2024b. Workforce Report 2024. General Medical Council.

[shil70250-bib-0021] General Medical Council . 2025. GMC Response to the Leng Review’s Call for Evidence Independent Review of Physician Associate and Anaesthesia Associate Professions. www.gmc‐uk.org/cdn/documents/gmc‐response‐to‐the‐leng‐review_pdf‐110582161.pdf.

[shil70250-bib-0022] Hafferty, F. W. , and D. W. Light . 1995. “Professional Dynamics and the Changing Nature of Medical Work.” Journal of Health and Social Behavior 35: 132–153 Special Issue. 10.2307/2626961. 7560845

[shil70250-bib-0023] Halter, M. , V. Drennan , K. Chattopadhyay , et al. 2013. “The Contribution of Physician Assistants in Primary Care: A Systematic Review.” BMC Health Services Research 13, no. 1: 223. 10.1186/1472-6963-13-223. 23773235 PMC3698179

[shil70250-bib-0024] Hooker, R. S. , and C. M. Everett . 2012. “The Contributions of Physician Assistants in Primary Care Systems.” Health and Social Care in the Community 20, no. 1: 20–31. 10.1111/j.1365-2524.2011.01021.x. 21851446 PMC3903046

[shil70250-bib-0025] Hooker, R. S. , and A. N. Muchow . 2015. “Modifying State Laws for Nurse Practitioners and Physician Assistants Can Reduce Cost of Medical Services.” Nursing Economics 33, no. 2: 88. 10.1097/01.NME.0000458612.58744.97. 26281279

[shil70250-bib-0026] House of Commons Library . 2024. Capacity Pressures in Health and Social Care in England. House of Commons Library.

[shil70250-bib-0027] Larson, M. S. 1977. The Rise of Professionalism: A Sociological Analysis. University of California Press.

[shil70250-bib-0028] Light, D. W. 2010. “Health‐Care Professions, Markets, and Countervailing Powers.” In Handbook of Medical Sociology, edited by C. E. Bird , P. Conrad , A. M. Fremont , and S. Timmermans , 6th ed., 270–289. Vanderbilt University Press.

[shil70250-bib-0029] McDermott, I. , J. Astbury , S. Jacobs , et al. 2023. “To Be or Not to Be: The Identity Work of Pharmacists as Clinicians.” Sociology of Health & Illness 45, no. 3: 623–641. 10.1111/1467-9566.13605. 36610016

[shil70250-bib-0030] McKinlay, J. B. , and L. D. Marceau . 2002. “The End of the Golden Age of Doctoring.” International Journal of Health Services 32, no. 2: 379–416. 10.2190/jl1d-21bg-pk2n-j0kd. 12067037

[shil70250-bib-0031] McMurray, R. 2011. “The Struggle to Professionalize: An Ethnographic Account of the Occupational Position of Advanced Nurse Practitioners.” Human Relations 64, no. 6: 801–822. 10.1177/0018726710387949.

[shil70250-bib-0032] Mittman, D. E. , J. F. Cawley , and W. H. Fenn . 2002. “Physician Assistants in the United States.” British Medical Journal 325, no. 7362: 485–487. 10.1136/bmj.325.7362.485. 12202333 PMC1124003

[shil70250-bib-0033] Nancarrow, S. A. , and A. M. Borthwick . 2005. “Dynamic Professional Boundaries in the Healthcare Workforce.” Sociology of Health & Illness 27, no. 7: 897–919. 10.1111/j.1467-9566.2005.00463.x. 16313522

[shil70250-bib-0034] NHS Employers . 2025. Physician Assistants. https://www.nhsemployers.org/articles/physician‐associates.

[shil70250-bib-0035] NHS England . 2023. NHS Long Term Workforce Plan. NHS England.

[shil70250-bib-0036] NHS England . 2024. Update on Physician Associates and Anaesthesia Associates. https://www.england.nhs.uk/long‐read/update‐on‐physician‐associates‐and‐anaesthesia‐associates/.

[shil70250-bib-0037] Noordegraaf, M. 2011. “Risky Business: How Professionals and Professional Fields (Must) Deal With Organizational Issues.” Organization Studies 32, no. 10: 1349–1371. 10.1177/0170840611416748.

[shil70250-bib-0038] Noordegraaf, M. 2020. “Protective or Connective Professionalism? How Connected Professionals Can (Still) Act as Autonomous and Authoritative Experts.” Journal of Professions and Organization 7, no. 2: 205–223. 10.1093/jpo/joaa011.

[shil70250-bib-0039] Parsons, T. 1951. “Illness and the Role of the Physician: A Sociological Perspective.” American Journal of Orthopsychiatry 21, no. 3: 452–460. 10.1111/j.1939-0025.1951.tb00003.x. 14857123

[shil70250-bib-0040] Potter, J. , and M. Wetherell . 1987. Discourse and Social Psychology: Beyond Attitudes and Behaviour. Sage.

[shil70250-bib-0041] Royal College of Physicians . 2024. Faculty of Physician Associates to Close in December 2024. https://www.rcp.ac.uk/news‐and‐media/news‐and‐opinion/faculty‐of‐physician‐associates‐to‐close‐in‐december‐2024/.

[shil70250-bib-0042] Saks, M. 2003. Orthodox and Alternative Medicine: Politics, Professionalization and Health Care. Sage.

[shil70250-bib-0043] Schot, E. , L. Tummers , and M. Noordegraaf . 2020. “Working on Working Together. A Systematic Review on How Healthcare Professionals Contribute to Interprofessional Collaboration.” Journal of Interprofessional Care 34, no. 3: 332–342. 10.1080/13561820.2019.1636007. 31329469

[shil70250-bib-0044] Suchman, M. C. 1995. “Managing Legitimacy: Strategic and Institutional Approaches.” Academy of Management Review 20, no. 3: 571–610. 10.2307/258788.

[shil70250-bib-0045] Swidler, A. 1986. “Culture in Action: Symbols and Strategies.” American Sociological Review 51, no. 2: 273–286. 10.2307/2095521.

[shil70250-bib-0046] Thompson, W. , and M. McNamara . 2022. “Revealing How Language Builds the Identity of the Advanced Nurse Practitioner.” Journal of Clinical Nursing 31, no. 15–16: 2344–2353. 10.1111/jocn.16054. 34561924

[shil70250-bib-0047] Timmermans, S. , and E. S. Kolker . 2004. “Evidence‐Based Medicine and the Reconfiguration of Medical Knowledge.” Journal of Health and Social Behavior 45, no. Extra Issue: 177–193. 10.2307/3653831. 15779473

[shil70250-bib-0048] Timmermans, S. , and H. Oh . 2010. “The Continued Social Transformation of the Medical Profession.” Supplement, Journal of Health and Social Behavior 51, no. Suppl: S94–S106. 10.1177/0022146510383500. 20943586

[shil70250-bib-0049] Timmons, S. , C. Mann , C. Evans , R. Pearce , C. Overton , and K. Hinsliff‐Smith . 2023. “The Advanced Clinical Practitioner (ACP) in UK Healthcare: Dichotomies in a New ‘Multi‐Professional’ Profession.” SSM‐Qualitative Research in Health 3: 100211. 10.1016/j.ssmqr.2022.100211.

[shil70250-bib-0050] UMAP . 2025a. Clinical Audit of Physician Associate Performance in OOH Settings in the United Kingdom: A Quantitative and Thematic Analysis.

[shil70250-bib-0051] UMAP . 2025b. Summary Report for the Leng Review: Evaluating Physician Associate Colleague Multi‐Source Feedback.

[shil70250-bib-0052] UMAP . 2025c. Summary Report for the Leng Review: Evaluating Physician Associate Patient Multi‐Source Feedback.

[shil70250-bib-0053] UMAP . 2025d. Summary Report for the Leng Review: Evaluating the Role of Physician Associates.

[shil70250-bib-0054] UMAP . 2025e. Summary Report for the Leng Review: Evaluating the Supervision of Physician Associates in Primary Care.

[shil70250-bib-0055] UMAP . 2025f. Summary Report for the Leng Review: Evaluating the Supervision of Physician Associates in Secondary Care.

[shil70250-bib-0056] UMAP . 2025g. Summary Report for the Leng Review: Significant and Never Events Involving Medical Associate Professionals.

[shil70250-bib-0057] UMAP . 2025h. Umaps & CMAPs Submits Landmark Evidence to Leng Review, Calls Out BMA for Misleading Narrative on MAPs. https://umaps.org.uk/umaps‐submits‐landmark‐evidence‐to‐leng‐review‐calls‐out‐bma‐for‐misleading‐narrative‐on‐maps.

[shil70250-bib-0058] UMAP . 2025i. Wellbeing Survey Report: Evaluating the Wellbeing of Physician Associates and Anaesthesia Associates.

[shil70250-bib-0059] Weiss, M. C. , and J. Sutton . 2009. “The Changing Nature of Prescribing: Pharmacists as Prescribers and Challenges to Medical Dominance.” Sociology of Health & Illness 31, no. 3: 406–421. 10.1111/j.1467-9566.2008.01142.x. 19055585

[shil70250-bib-0060] Wetherell, M. , and J. Potter . 1988. “Discourse Analysis and the Identification of Interpretative Repertoires.” Analysing Everyday Explanation: A Casebook of Methods: 168–183.

[shil70250-bib-0061] Williams, L. E. , and T. S. Ritsema . 2014. “Satisfaction of Doctors With the Role of Physician Associates.” Clinical Medicine 14, no. 2: 113–116. 10.7861/clinmedicine.14-2-113. 24715119 PMC4953279

[shil70250-bib-0062] Willis, E. 1989. Medical Dominance: The Division of Labour in Australian Health Care. Allen & Unwin.

[shil70250-bib-0063] Witz, A. 1992. Professions and Patriarchy. Routledge.

